# Inclement weather forces stopovers and prevents migratory progress for obligate soaring migrants

**DOI:** 10.1186/s40462-021-00274-6

**Published:** 2021-07-10

**Authors:** Julie M. Mallon, Keith L. Bildstein, William F. Fagan

**Affiliations:** 1 Department of Biology, University of Maryland, College Park, MD 20742 USA; 2 Hawk Mountain Sanctuary, Acopian Center for Conservation Learning, 410 Summer Valley Road, Orwigsburg, PA 17961 USA

**Keywords:** Flight behavior, Fly-and-forage, Raptors, Cathartidae, Energy minimization, Soaring, Track annotation

## Abstract

**Background:**

Migrating birds experience weather conditions that change with time, which affect their decision to stop or resume migration. Soaring migrants are especially sensitive to changing weather conditions because they rely on the availability of environmental updrafts to subsidize flight. The timescale that local weather conditions change over is on the order of hours, while stopovers are studied at the daily scale, creating a temporal mismatch.

**Methods:**

We used GPS satellite tracking data from four migratory Turkey Vulture (*Cathartes aura*) populations, paired with local weather data, to determine if the decision to stopover by migrating Turkey Vultures was in response to changing local weather conditions. We analyzed 174 migrations of 34 individuals from 2006 to 2019 and identified 589 stopovers based on variance of first passage times. We also investigated if the extent of movement activity correlated with average weather conditions experienced during a stopover, and report general patterns of stopover use by Turkey Vultures between seasons and across populations.

**Results:**

Stopover duration ranged from 2 h to more than 11 days, with 51 % of stopovers lasting < 24 h. Turkey Vultures began stopovers immediately in response to changes in weather variables that did not favor thermal soaring (e.g., increasing precipitation fraction and decreasing thermal updraft velocity) and their departure from stopovers was associated with improvements in weather that favored thermal development. During stopovers, proportion of activity was negatively associated with precipitation but was positively associated with temperature and thermal updraft velocity.

**Conclusions:**

The rapid response of migrating Turkey Vultures to changing weather conditions indicates weather-avoidance is one of the major functions of their stopover use. During stopovers, however, the positive relationship between proportion of movement activity and conditions that promote thermal development suggests not all stopovers are used for weather-avoidance. Our results show that birds are capable of responding rapidly to their environment; therefore, for studies interested in external drivers of weather-related stopovers, it is essential that stopovers be identified at fine temporal scales.

**Supplementary Information:**

The online version contains supplementary material available at 10.1186/s40462-021-00274-6.

## Background

As birds migrate, they pass through a variety of habitats and experience variable environmental conditions. Local weather conditions in these habitats change with time, which influence the flight behavior of migrants, especially the likelihood of suspending and resuming active migration [1, 2]. Passerines avoid departing stopovers during precipitation [3] and are more likely to resume active migration during low wind speeds and decreasing surface pressure [4]. Migratory raptors also avoid migrating during poor weather conditions; they are rarely observed migrating during cold fronts but are observed in peak numbers immediately following a passing cold front [5, 6]. Similarly, the likelihood of terrestrial soaring birds stopping over increases on days with cloud cover and rain [7] and when thermal updraft strength is weak (*sensu* [8]).

Soaring birds are especially sensitive to changing weather conditions because they rely on the availability of environmental updrafts to subsidize flight. Although terrestrial birds can soar using orographic updrafts [9] or turbulence [10] to subsidize flight, thermals [8] are the most important type of updraft for most soaring migrants because thermals are widely distributed across the landscape [11], allow birds to reach altitudes necessary for fast cross-country soaring, and allow for straight, efficient flight paths [12]. Thermals (i.e., vortices of ascending hot air surrounded by descending cooler air) [11, 13] are generated by differential heating of the earth’s surface. Thermals are, therefore, an uncertain resource that only form under appropriate weather conditions [9, 13]. Poor weather conditions slow or prevent the development of updrafts, thereby forcing birds to switch to energetically expensive flapping flight [14, 15] or grounding them.

Despite recognizing the relationship between migratory flight behavior and changing weather conditions, stopover use by large, soaring birds has been understudied. This is in part due to challenges around defining what constitutes a stopover - rule selection varies depending on the species ecology and desired behavior. Some of the definitions of stopovers for raptors include: ≤ 150 km/day [16], < 100 km/day [17, 18], < 50 km/day of directed flight [19], ≤ 25 km/day [20], > 24 h in an area < 30 km in diameter [21], and spending more than 24 h in an area [22]. These definitions pose two problems. First, all of these definitions consider the durations of stopovers to be ≥ 24 h, due in part to the assumption that stopovers are used primarily to refuel. If we define stopovers based on the movement behavior of individuals, however, stopovers occur whenever individuals do not engage in fast, directed flight but are otherwise expected to do so. Using this movement-based definition of stopovers, the duration of stopovers may be much shorter than 24 h as local weather conditions can change rapidly and may only prevent birds from flying for short periods of time (i.e., hours). Clearly, standardized measures that constitute what are and are not stopovers are warranted.

Stopover behavior is ideal to study using Turkey Vultures (*Cathartes aura*) because they are obligate soaring migrants [23] and cannot sustain themselves aloft for long periods using flapping flight [24]. Hence, Turkey Vultures should be sensitive to changes in weather and stop frequently to avoid poor weather conditions. Here we investigate if stopovers used by Turkey Vultures are linked to changes in weather conditions that are poor for thermal soaring. To this end, we used satellite tracking data from four migratory Turkey Vulture populations, paired with local weather data. As a first step, we identified stopovers using first passage time (FPT) [25]. We automated identification of stopovers by selecting the radius and FPT threshold based on the structure of the data to avoid under- and over-selection of stopovers. We explored general patterns of stopover use by comparing the frequencies of stopover use between seasons and across populations.

Next, we evaluated when Turkey Vultures began their stopovers, relative to changing local weather conditions. As obligate scavengers, Turkey Vultures have several behavioral and physiological adaptations to minimize energetic costs [10, 26, 27]. We hypothesized that, to further minimize energetic costs, Turkey Vultures should fly as long as weather conditions allow for energy-efficient soaring flight and would stop as soon as weather conditions deteriorate, rather than switch to flapping flight [28, 29]. Last, we evaluated if average weather conditions experienced by Turkey Vultures also affected their general movement activity during stopovers.

## Methods

### Study species

We used GPS-GSM tracking data collected between 2006 and 2019 from four migratory populations that represent three of the seven subspecies of Turkey Vulture (*C. aura aura, C. aura meridionalis, and C. aura ruficollis*). These populations range across most of the species’ distribution – from Canada to southern South America (Supplemental Fig. 1). Tracking data were provided by Hawk Mountain Sanctuary (Pennsylvania, USA) and accessed via Movebank [30].

### Weather predictors

We accessed weather data using Movebank Env-data annotation [31]. We selected weather variables that were known to either be important for thermal soaring and orographic soaring or were related to inclement weather (Supplemental Table 1). To focus on the behavioral response of Turkey Vultures to changing weather conditions, we excluded any static variables (e.g. NDVI, landcover).

### Stopover classification

We used only migrations with regularly collected data (i.e., mean data interval < 3 h). We annotated migrations from continuous tracks using first passage time (FPT; R package adehabitatLT) [32] and manually reviewed each migration for precision of start and end dates. FPT measures the time it takes an individual to leave a circle of a given radius, which informs about an individual’s behavioral state. Short FPTs indicate straight, fast movements such as commuting between habitats [33] or migration [34]. Long FPTs indicate localized movements such as foraging [25, 33, 35], residency [34], or resting [35].

For the purposes of annotating stopover locations, we linearly interpolated any spatiotemporal gaps within the movement trajectories to get hourly fixes. To find which days potentially included stopovers using FPT, we first selected the radius that maximized log-variance of FPT (between 2500 and 6000 m) for each migration. We selected this range of radii based on patterns of activity and inactivity in our dataset, which accounted for different movement speeds among populations and between seasons.

We anticipated some stopovers may be quite short because Turkey Vultures are diurnal migrants. For example, if weather fronts moved in during the late afternoon (i.e., flight hours) and passed overnight (i.e., roosting hours) a Turkey Vulture may stopover, ceasing flight before their normal roosting hours, but such a stopover is likely to go undetected by normal estimates [16–22]. Therefore, we chose to be highly conservative with our selection of stopovers and identified the start and end of stopovers at the hourly scale.

To find the start and end times of each stopover, we used a threshold relative to the variance of the FPT for each migration; this reduced under-selection of stopovers from tracks with high FPT variance and over-selection of stopovers from tracks with low FPT variance. Stopovers selected from FPT were rejected if < 25 % of the data were during daylight hours or if the duration was < 2 h. To find non-stationary stopovers that were missed with this approach, we considered groups of > 30 points within a 15 km buffer to be a ‘stopover’ (where the mean duration of 30 points in our dataset is 41 ± 10 h). To improve the precision of the start and end times of stopovers in our dataset, we removed the first and last observations if those speeds exceeded 95 % of all the speeds during the stopover. If an individual ceased activity during normal roosting hours (approx. 1700–0800 h), we considered 0800 the following morning to be the start of the stopover.

### Analysis

To determine if there was a difference in the frequency of stopover use among populations or between seasons, we used a linear mixed model. The response variable was the total number of stopovers per migration, the fixed effects were population, season, and the interaction of population and season, and the random effect was individual id.

To determine if Turkey Vultures used stopovers in response to changes in weather at the species level, we plotted the average hourly changes for each weather variable. As not all stopovers were expected to be in response to weather, we sought to remove noise from the dataset by first ranking stopovers by their proportion of activity. Proportion of activity, or the proportion of daytime hours where there was flight activity, was calculated for non-roosting hours, which were determined by local sunrise and sunset times. To ensure the movement activity represented flight activity, rather than geolocation errors, we defined activity as > 1 km/h. To avoid including stopovers used for feeding, we excluded the most active third of stopovers from our dataset (*n* = 189) for these analyses. We subset each weather variable from 7 h before to 7 h after the start of each stopover. We selected a 14 h window to avoid signals related to normal diurnal patterns in weather, while also capturing the full range of flight hours of a diurnal migrant. To facilitate comparison across populations with different climates and across years, we used the hourly change of each weather parameter as our response variables. For each hour, we calculated the rate of change across all stopovers. To account for uneven numbers of stopovers among individuals, we averaged these hourly values to the individual level. For each variable, we used a loess smoother to visually inspect the average rate of change over time relative to the start of stopovers. We then determined if the start of stopovers lagged relative to the minima and maxima for each weather variable. To determine if the decision to depart from stopovers lagged relative to changing environmental conditions, we repeated this analysis for the end of stopovers. We report the population-specific responses to changing weather variables at the start and end of stopovers in the Supplementary materials.

To compare movement behavior during stopovers, we used proportion of activity as the response variable. To evaluate the effect of mean weather conditions during a stopover on movement behavior, we used a binomial generalized linear mixed model (GLMM) to identify if any weather variables were associated with daytime movement during stopovers. Our response variable was the proportion of active hours out of the number of daytime hours during the stopover. We used only stopovers with complete weather data (*n* = 460) and used the mean of each weather variable (Supplemental Table 1) during the stopover as a predictor in our full model. We removed any predictors with correlations > 0.6, retaining the predictor that we expected to be the most ecologically relevant. We included individuals nested within population as our random effect. All analyses were done using R version 4.0.2 [36].

## Results

### Description of stopover use

We analyzed 174 migrations of 34 individuals from 2006 to 2019 and identified 589 stopovers. Frequency of stopover use varied among populations and between seasons (Table 1). Regardless of season, the Central Canada population used stopovers most frequently (β = 6.159, SE = 0.967, t = 6.372; Supplemental materials). All populations except Western Canada used stopovers more frequently during fall migration than during spring migration (β = 1.938, SE = 1.000, t = 1.938; Supplemental materials).

**Table 1:** Seasonal differences in stopover use among populations. We report the total, mean, and standard error of the number of stopovers used by each population and during each migration season. As stopover use is expected to vary as a function of migration distance, we report the mean and standard error of migration distance per stopover used

Season	Population	Individuals	Total number of migrations	Total number of stopovers	Number of stopovers per migration (mean ± se)	Migration distance (km, mean ± se)
Spring	Southwest USA	14	39	44	1.1 ± 0.2	2690 ± 163.6
	Central Canada	6	19	128	6.7 ± 0.7	1409 ± 297.2
	Western Canada	6	11	32	2.9 ± 0.4	1371 ± 220.3
	Southern South America	4	11	22	2.0 ± 0.5	1735 ± 232.3
Fall	Southwest USA	14	45	102	2.3 ± 0.3	1996 ± 171.9
	Central Canada	8	23	188	8.2 ± 0.9	960 ± 105.7
	Western Canada	6	11	22	2.0 ± 0.6	2187 ± 283.9
	Southern South America	5	15	51	3.4 ± 0.7	1382 ± 212.8

**Fig. 1:**
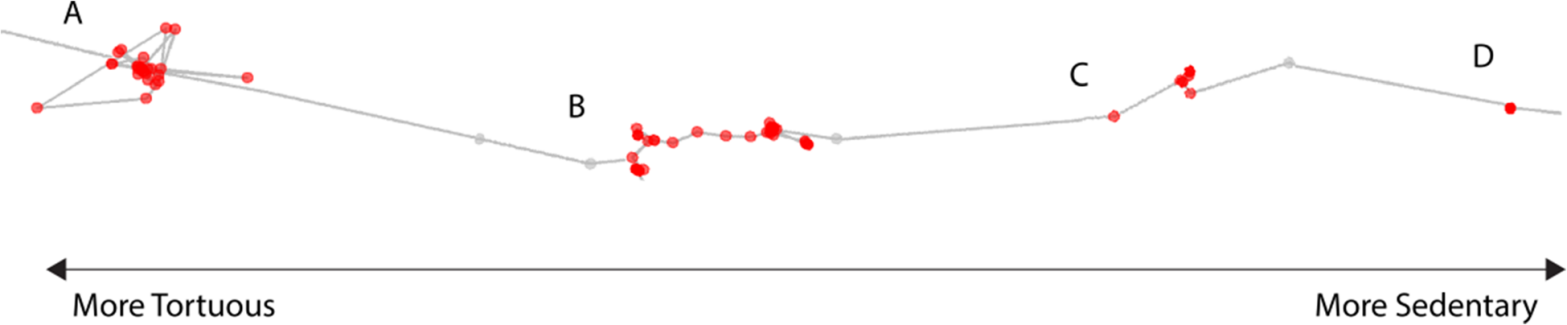
A composite of four stopovers (red) ranked from most tortuous (**A**) to most sedentary (**D**). Active migration is shown in gray. For small radii,  more tortuous stopovers have lower first passage times and higher proportions of activity, while more sedentary stopovers have higher first passage times and lower proportions of activity

Movement behavior during stopovers varied from highly sedentary to highly tortuous (Fig. 1). Total distances moved during stops ranged from 0 to 601 km. Duration of stopovers ranged from 2 h to more than 11 days. 51 % of stopovers (*n* = 300) were < 24 h in duration. 58 % of stopovers (*n* = 342) started or ended on a day where birds migrated > 100 km. All stopovers > 24 h (*n* = 289) had at least one stopover day where the bird travelled a total distance < 100 km. Of these, 90 % (*n* = 260) had at least one stopover day where the bird travelled a total distance < 25 km.

### Onset of stopovers

**Fig. 2:**
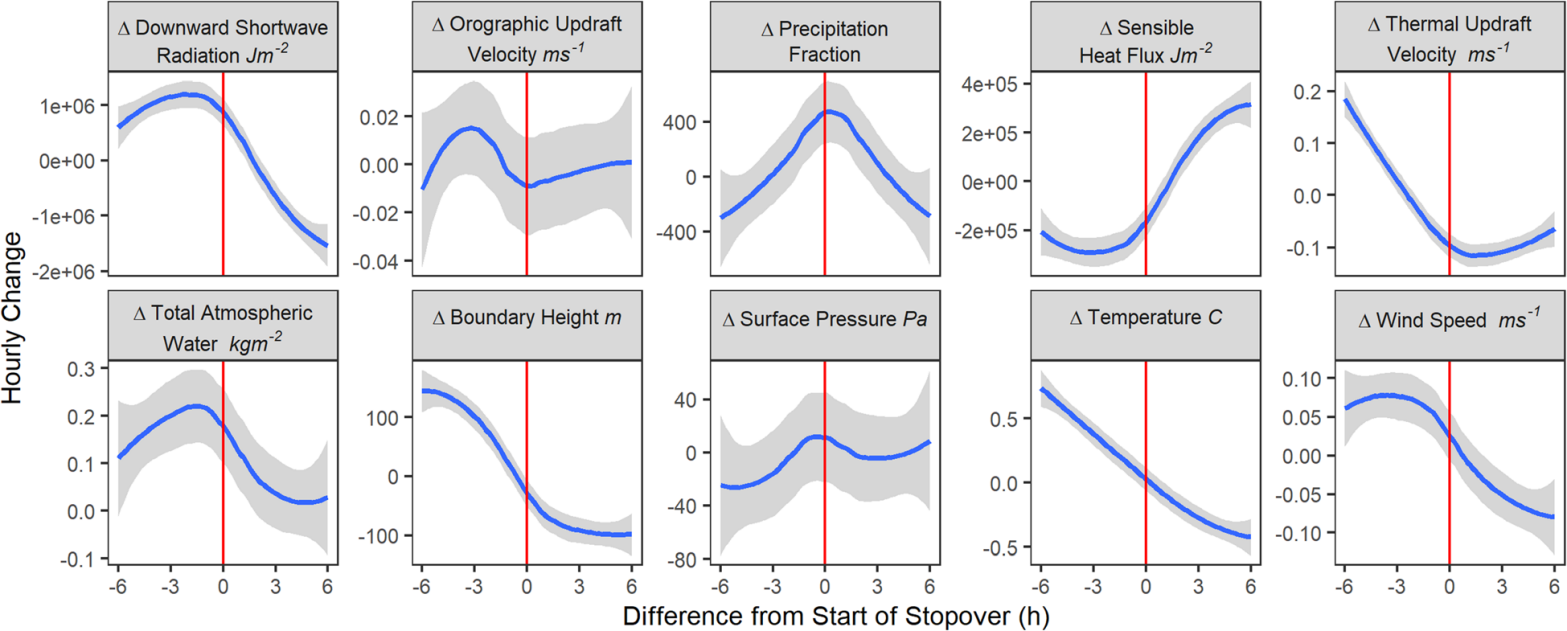
Weather conditions relative to the start of *n* = 395 stopovers (red line), averaged to individual birds (*n* = 34). The y-axis represents the hourly change of the variable indicated in each plot’s title. The average hourly change in each weather variable is shown in blue and the 95 % confidence interval around this estimate is shown in gray. Peaks of several variables (i.e., downward shortwave radiation, precipitation fraction, sensible heat flux, and total atmospheric water) are within one hour of the start of stopover, indicating rapid response by Turkey Vultures to deteriorating weather conditions. Several other variables are declining at the start of stopover, i.e., thermal updraft velocity, boundary height, temperature, and wind speed

Stopovers typically began in the midafternoon (Supplemental Fig. 2). At the species-level, the beginning of stopovers temporally matched the peak rate of change of precipitation fraction (Fig. 2). Several weather variables (Supplemental Table 1) had peak rates of change within one to two hours of the start of stopovers, including downward shortwave radiation, sensible heat flux, thermal updraft velocity, and total atmospheric water (Fig. 2).

There was no predictable response to change in surface pressure or orographic updraft velocity. Other weather variables showed no peak in the rate of change but a gradual change over time, including boundary height, temperature, and wind speed. Responses were similar across all populations (Supplemental materials).

### Departure from stopovers

**Fig. 3:**
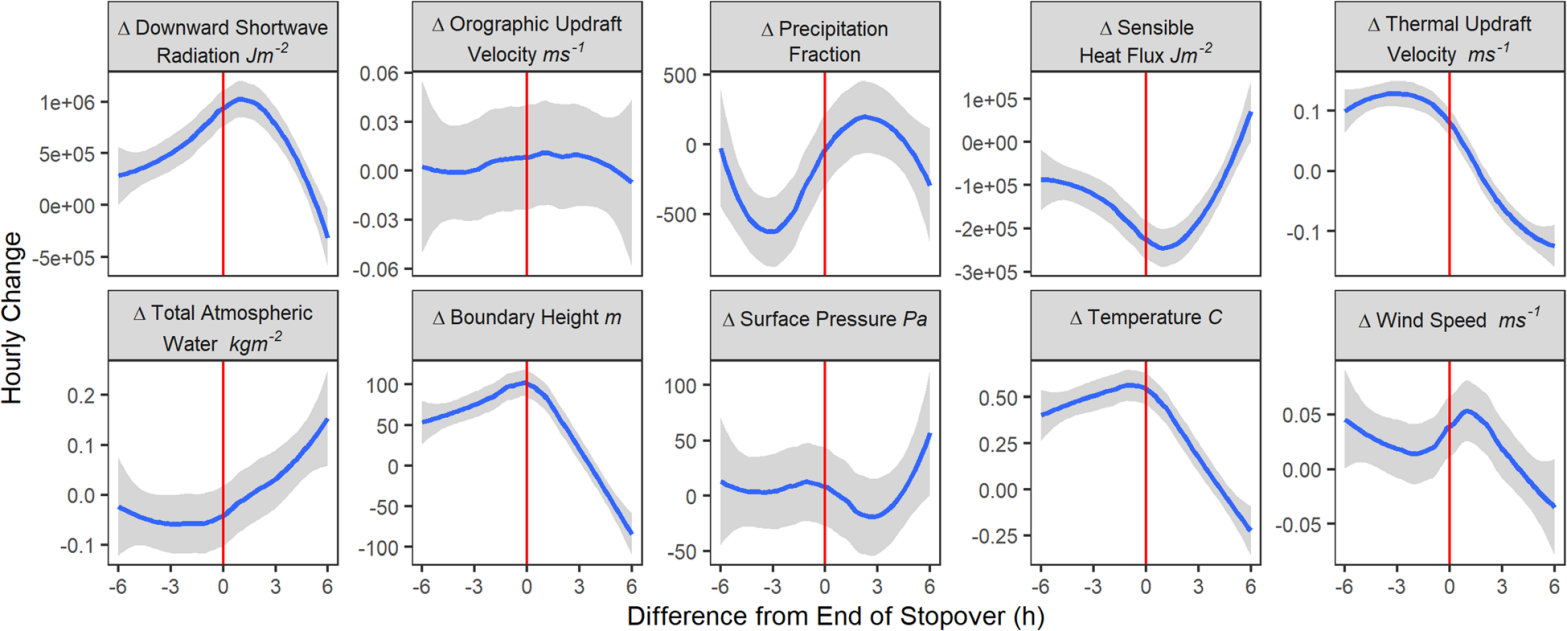
Weather conditions relative to the end of *n* = 395 stopovers (red line), averaged to individual birds (*n* = 34). The y-axis represents the hourly change of the variable indicated in each plot’s title. The average hourly change in each weather variable is shown in blue and the 95 % confidence interval around this estimate is shown in gray. Peaks of boundary height and temperature are within one hour of the end of stopover, and several other peaks are within three hours of the end of the stopover, indicating a response by Turkey Vultures to improving weather conditions

Stopovers typically ended in the early morning (Supplemental Fig. 2). At the species-level, Turkey Vultures showed a response to fewer weather variables when departing stopovers and resuming migration (Fig. 3). Stopover departures were delayed relative to the peak rate of change of thermal updraft velocity but temporally matched the peak rates of change for temperature and boundary height. In contrast, the rate of change in downward shortwave thermal radiation peaked more than one hour after the end of stopovers.

Departure from stopovers also followed decreases in the rate of change of precipitation fraction and sensible heat flux. There was no predictable response to change in orographic updraft velocity, total atmospheric water, surface pressure, or wind speed. Responses were similar across three of four populations; however, the Western Canada population departed from stopovers during increasing rates of precipitation (Supplemental Fig. 4c).

### Movement behavior during stopovers

Movement behavior during stopovers was associated with weather conditions that promoted thermal soaring (Table 2). Proportion of activity during stopovers increased with mean thermal updraft velocity and mean temperature but decreased with mean precipitation fraction (Table 2).

**Table 2:** Coefficients of the top generalized mixed-effects model (GLMM; binomial) using mean values of weather variables to predict the proportion of activity during stopovers (where a bird moved > 1 km per hour) by GPS-GSM tagged Turkey Vultures (*n* = 460). Individual (*n* = 34) nested within population (*n* = 4) was included as a random effect (both random effects: SD = 0.079). Estimates reported here are of unscaled predictors

Variable	β	SE	z	*p*-value
Intercept	-1.7332	0.1082	-4.759	< 0.001
Precipitation Fraction	-3.7785	0.1235	-2.348	0.0189
Thermal Updraft Velocity	9.2448	0.1050	2.231	0.0257
Temperature	2.9703	0.1224	3.306	< 0.001

## Discussion

We found that Turkey Vultures frequently stopped actively migrating to avoid flying during poor weather conditions, adding to the growing literature recognizing weather avoidance as an important driver of stopover use by migrating birds [37–39]. Turkey Vultures ceased directed flight quickly in response to high rates of change in several weather variables and resumed migration when conditions improved. As obligate soaring birds, vultures are highly sensitive to changing weather conditions that affect the availability of updrafts. The variables they responded to are broadly associated with thermal updraft strength [40, 41], which is the most important type of updraft for these migratory populations [9]. The lack of response to orographic updraft velocity suggests that this was not an important updraft source for these populations, which largely avoid areas of high topographic relief along their migration routes.

Boundary layer, temperature, and thermal updraft velocity all follow a diurnal pattern [42] that may partly explain the close response to these variables by Turkey Vultures. The most frequent time for take-off for vultures to resume migration was in the morning. At early hours, there is rapid change in variables such as temperature and solar radiation, which lead to the development of thermals. Likewise, the most frequent time for the start of stopovers was in the afternoon when there is a slow decline in thermal strength and an increase in sensible heat flux [9]. The positive relationships between these variables and active migration is consistent with the behaviors of other soaring birds. Increased temperatures are associated with departure from staging sites by Bee-eaters *Merops apiaster* [43]. Boundary layer height is associated with faster migration speeds of Montagu’s Harriers *Circus pygargus* and Honey Buzzards *Pernis apivorus* [20] and proportion of time spent soaring by Lesser Black-backed Gulls *Larus fuscus* [44]. Importantly, we found that the onset and cessation of stopovers by Turkey Vultures are associated with high rates of change in weather. The effect of the magnitude of change of each weather variable on the stopover behavior of vultures requires further study.

Increases in variables that do not fluctuate diurnally, such as precipitation, can disrupt updrafts and slow progress for some soaring migrants [20]. However, the onset and cessation of stopovers used by Turkey Vultures was not associated with wind speed. This finding echoes results for Ospreys *Pandion haliaetus*, which are also soaring migrants that frequently use stopovers: Ospreys’ decisions to switch between active migration and stopover are not influenced by winds [45]. Another variable that we did not detect a response by Turkey Vultures to was surface air pressure. Although longer stopovers may have been associated with changes in surface air pressure, the time scale of our analyses was too fine to detect a response to changes in surface air pressure [43].

### Short stopovers

Identifying stopovers at the daily scale may underestimate the duration of stopovers that are > 24 h. For example, birds may actively migrate for several hours before stopping over and thereby exceed the distance threshold. The following day would be identified as the start of the stopover, several hours after the bird ceased directed flight. Half of the stopovers we identified did not meet the < 100 km and > 24 h criteria commonly used by other studies [16–22]. Only 4 % of stopovers we identified started and ended during normal roosting hours (approx. 1700–0800 h), where the timing of movement activity would match with the normal flight activity patterns of diurnal migrants [46]. Stopovers identified at the daily scale, thus, would have incorrectly identified the start and end times for 96 % of stopovers. As revealed by our results, birds are capable of responding rapidly to their environment; therefore, for studies interested in external drivers of stopover use, it is essential that stopovers be identified at fine temporal scales.

As identification of stopovers at the daily scale results in underestimates of stopover use, other soaring migrants may be shown to use short duration stopovers frequently. Several studies of migrating raptors only report results on stopovers > 24 h in duration, including: Swainson’s Hawks *Buteo swainsoni* [16], Osprey [17, 18, 22], Black Kites *Milvus migrans* [19], Montagu’s Harriers [20], Honey-buzzards [20, 21], Grey-faced Buzzards *Butastur indicus* [21], and Egyptian Vultures *Neophron percnopterus* [47, 48]. Given that half of the stopovers identified in our study were less than 24 h, a reanalysis of stopover use at finer temporal scales is warranted.

Stopover use among migrants that forage en route is likely to be underestimated using existing definitions of stopovers. A migrant that is refueling may travel tens of kilometers in a day searching for food and would exceed these distance-based stopover thresholds even though they are not engaged in directed migratory flight. Several raptor species, including Montagu’s Harriers [49], Ospreys [50] and Eleonora’s Falcons *Falco eleonorae* [51], feed while migrating. Feeding en route results in lower daily migration distances [45] and in slower, more tortuous movements [51], but allows migrants to continue in the direction of their goal.

We found several stopovers where vultures were slow moving (see Fig. 1B) that suggest individuals were searching for food en route. However, these same stopovers could be the result of Turkey Vultures that are adverse to stopping, instead attempting to migrate while its progress was slowed by weak updrafts. Although Turkey Vultures are expected to complete most of their migrations while fasting [52], the relationship between movement activity during stopovers and weather conditions that promote the development of thermals also suggests not all stopovers are weather-related and some vultures do stop to feed during migration. The rates that stopovers are used for refueling versus weather-avoidance requires further study.

## Conclusions

We sought to determine if stopover use by migrating Turkey Vultures was associated with changes in local weather conditions. We used a data-driven approach to identify short-duration stopovers < 24 h, which are used as frequently as stopovers > 24 h. The decision for Turkey Vultures to stop and resume active migration was in direct response to changes in several weather variables associated with thermal soaring, indicating that avoidance of poor weather conditions is a major function of stopover use by Turkey Vultures. Movement behavior during stopovers was typically driven by local weather conditions, where individuals moved more frequently during conditions that promote thermal updraft development, possibly in search of carrion. Whether or not such behavior occurs in other soaring migrants awaits additional study.

## Supplementary Information

**Additional file 1: Supplemental Figure 1.** Map of migrations by 34 individuals from four populations that represent three subspecies: Southwest USA (orange; C. aura aura), Central Canada (purple; C. aura meridionalis), Western Canada (dark green; C. aura meridionalis), and Southern South America (light green; C. aura ruficollis). White points indicate stopover locations (n = 589). **Supplemental Figure 2.** Histogram plots of the hours of the starts and ends of stopovers. Stopovers started most frequently around 1300–1500 h and ended most frequently around 1100 h. Typical roosting times begin and end approximately at 1700 and 0800 h, respectively. Due to some gaps in the data, some stopovers appeared to end during normal roosting hours (i.e., before 0700 h or after 1700 h). **Supplemental Figure 3.** Average weather conditions for each individual, relative to the start of identified stopovers (red line), by population. The y-axis represents the hourly change of the variable indicated in each plot’s title. Individuals in each population: (a) Southwest USA *n* = 14, (b) Central Canada *n* = 9, (c) Western Canada *n* = 6, and (d) Southern South America *n* = 5. Differences in responses across populations may be explained by unequal sample sizes, differing weather variable interactions associated with local climates, and stopovers used for feeding. **Supplemental Figure 4.** Average weather conditions for each individual, relative to the end of identified stopovers (red line), by population. The y-axis represents the hourly change of the variable indicated in each plot’s title. Individuals in each population: (a) Southwest USA *n* = 14, (b) Central Canada *n* = 9, (c) Western Canada *n* = 6, (d) Southern South America *n* = 5. Differences in responses across populations may be explained by unequal sample sizes, differing weather variable interactions associated with local climates, and stopovers used for feeding. **Supplemental Table 1.** Definitions of weather variables used and the rationale for including these variables. For variables that have units, units are provided in parentheses. Weather data were sourced from European Centre for Medium-Range Weather Forecasts (ECMWF) or Movebank. **Supplemental Table 2.** Linear mixed model summary of the number of stops per migration by population and season. Model was fit using lme4 package version 1.1.23. **Supplemental Table 3.** Linear mixed model summary of the number of stops per migration by *total migration distance*. Model was fit using lme4 package version 1.1.23.

## Data Availability

The dataset supporting the conclusions of this article is available in the Zenodo repository, 10.5281/zenodo.4271905. The original dataset analyzed during the current study are available in the Movebank Data Repository, 10.5441/001/1.f3qt46r2 [30].

## Abbreviations

FPT: First passage time
NDVI: Normalized difference vegetative index
GLMM: Generalized linear mixed model
