# Supplementary material for: Inclement weather forces stopovers and prevents migratory progress for obligate soaring migrants
Source: Mov Ecol. 2021 Jul 10;9:39. doi: 10.1186/s40462-021-00274-6 (PMC8272267; doi:10.1186/s40462-021-00274-6)
Supplement: Supplementary file 1 — Additional file 1: Supplemental Figure 1. Map of migrations by 34 individuals from four populations that represent three subspecies: Southwest USA (orange; C. aura aura), Central Canada (purple; C. aura meridionalis), Western Canada (dark green; C. aura meridionalis), and Southern South America (light green; C. aura ruficollis). White points indicate stopover locations (n = 589). Supplemental Figure 2. Histogram plots of the hours of the starts and ends of stopovers. Stopovers started most frequently around 1300–1500 h and ended most frequently around 1100 h. Typical roosting times begin and end approximately at 1700 and 0800 h, respectively. Due to some gaps in the data, some stopovers appeared to end during normal roosting hours (i.e., before 0700 h or after 1700 h). Supplemental Figure 3. Average weather conditions for each individual, relative to the start of identified stopovers (red line), by population. The y-axis represents the hourly change of the variable indicated in each plot’s title. Individuals in each population: (a) Southwest USA n = 14, (b) Central Canada n = 9, (c) Western Canada n = 6, and (d) Southern South America n = 5. Differences in responses across populations may be explained by unequal sample sizes, differing weather variable interactions associated with local climates, and stopovers used for feeding. Supplemental Figure 4. Average weather conditions for each individual, relative to the end of identified stopovers (red line), by population. The y-axis represents the hourly change of the variable indicated in each plot’s title. Individuals in each population: (a) Southwest USA n = 14, (b) Central Canada n = 9, (c) Western Canada n = 6, (d) Southern South America n = 5. Differences in responses across populations may be explained by unequal sample sizes, differing weather variable interactions associated with local climates, and stopovers used for feeding. Supplemental Table 1. Definitions of weather variables used and the rationale for incl [file 40462_2021_274_MOESM1_ESM.docx]

# Supplementary Material


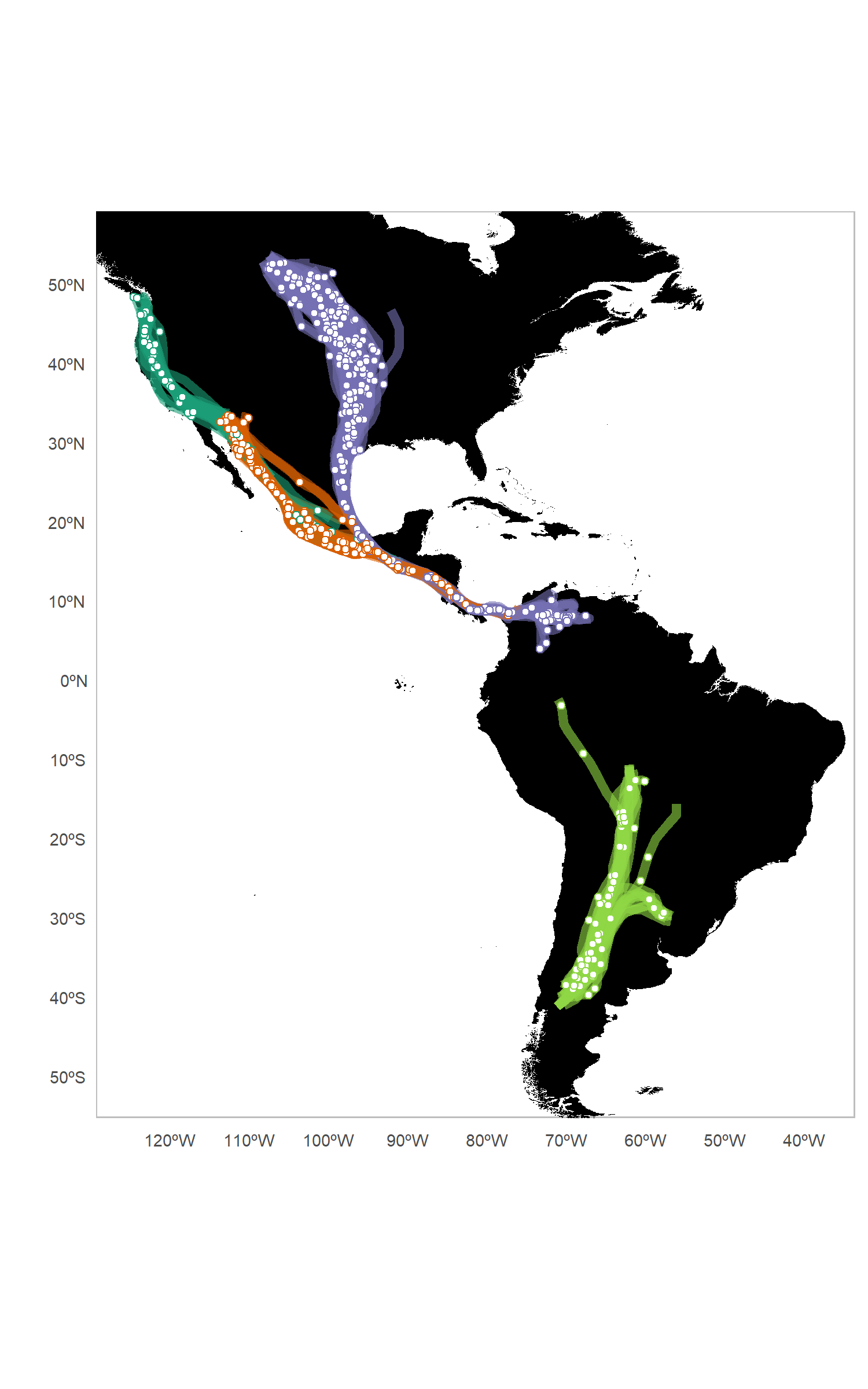

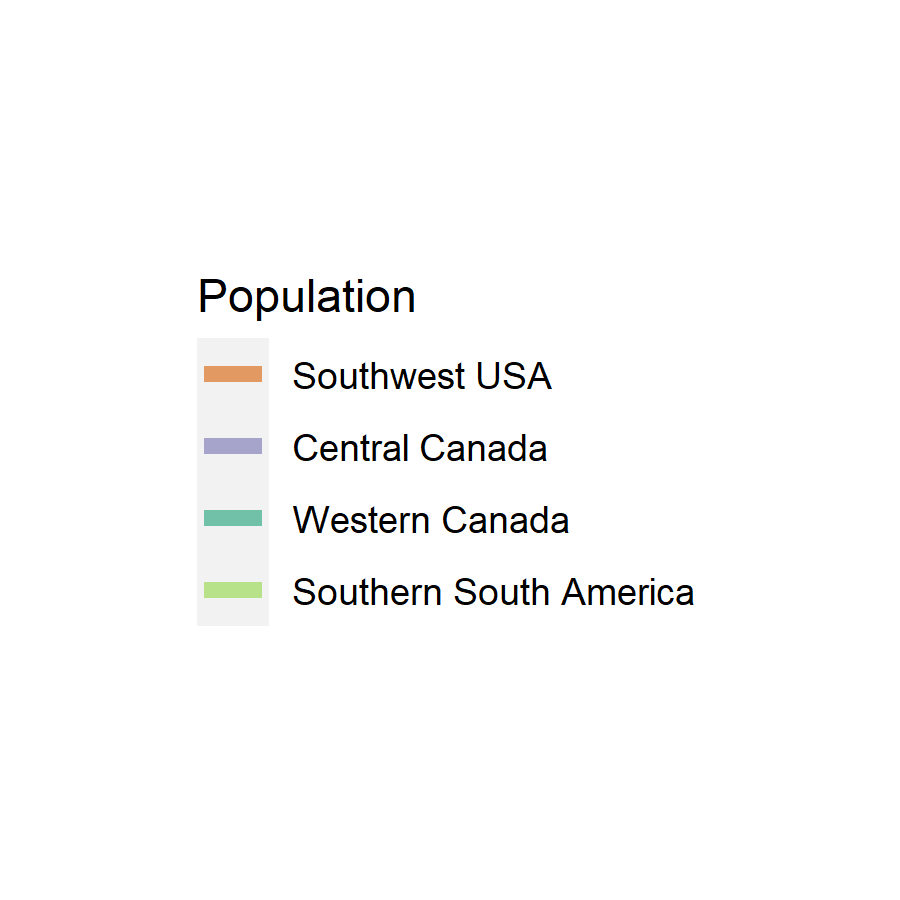


**Supplemental Figure 1.** Map of migrations by 34 individuals from four populations that represent three subspecies: Southwest USA (orange; *C. aura aura*), Central Canada (purple; *C. aura meridionalis*), Western Canada (dark green; *C. aura meridionalis*), and Southern South America (light green; *C. aura ruficollis*). White points indicate stopover locations (n = 589).

**
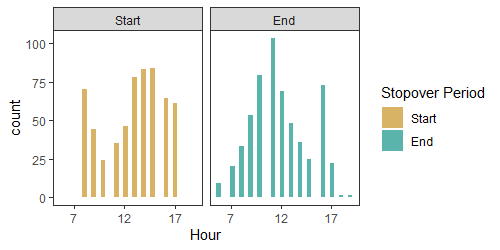
**

**Supplemental Figure 2**. Histogram plots of the hours of the starts and ends of stopovers. Stopovers started most frequently around 1300-1500 h and ended most frequently around 1100 h. Typical roosting times begin and end approximately at 1700 h and 0800 h, respectively. Due to some gaps in the data, some stopovers appeared to end during normal roosting hours (i.e., before 0700 h or after 1700 h).

 (a) Southwest USA


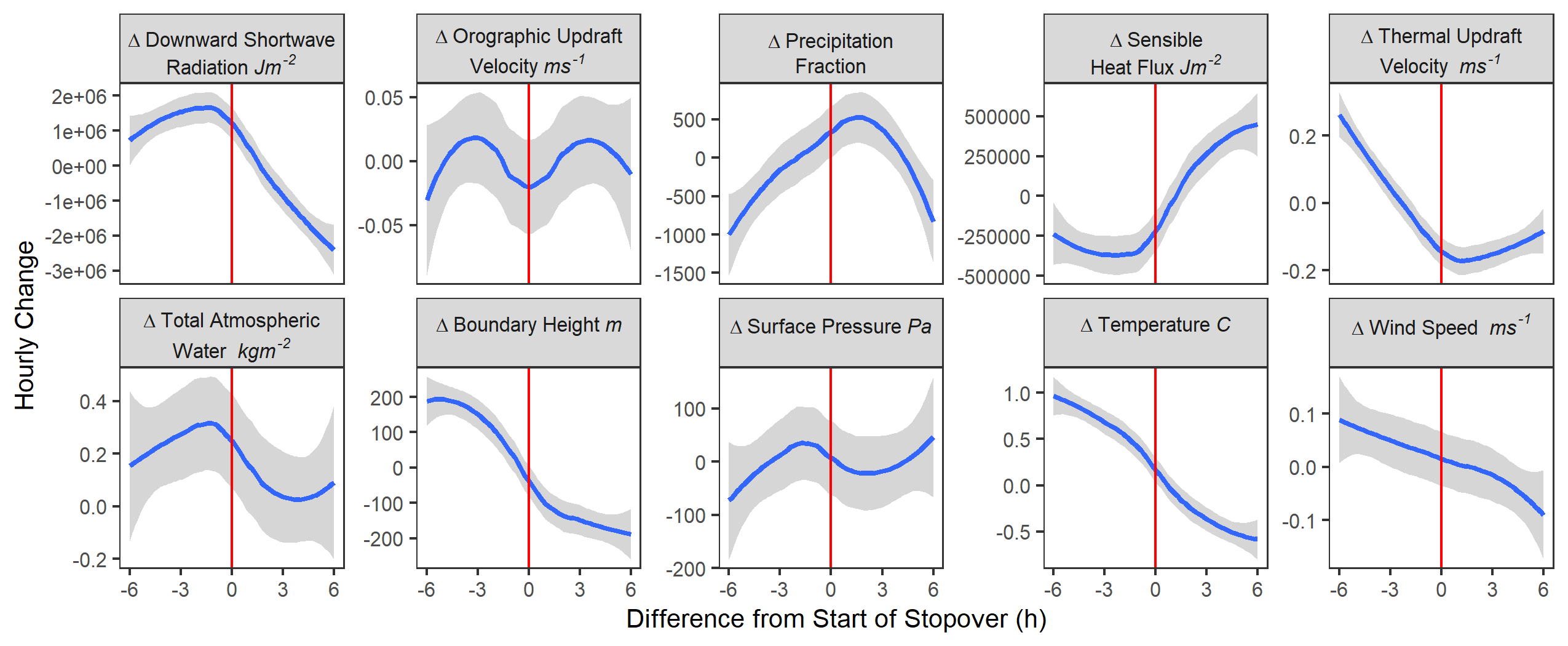


(b) Central Canada


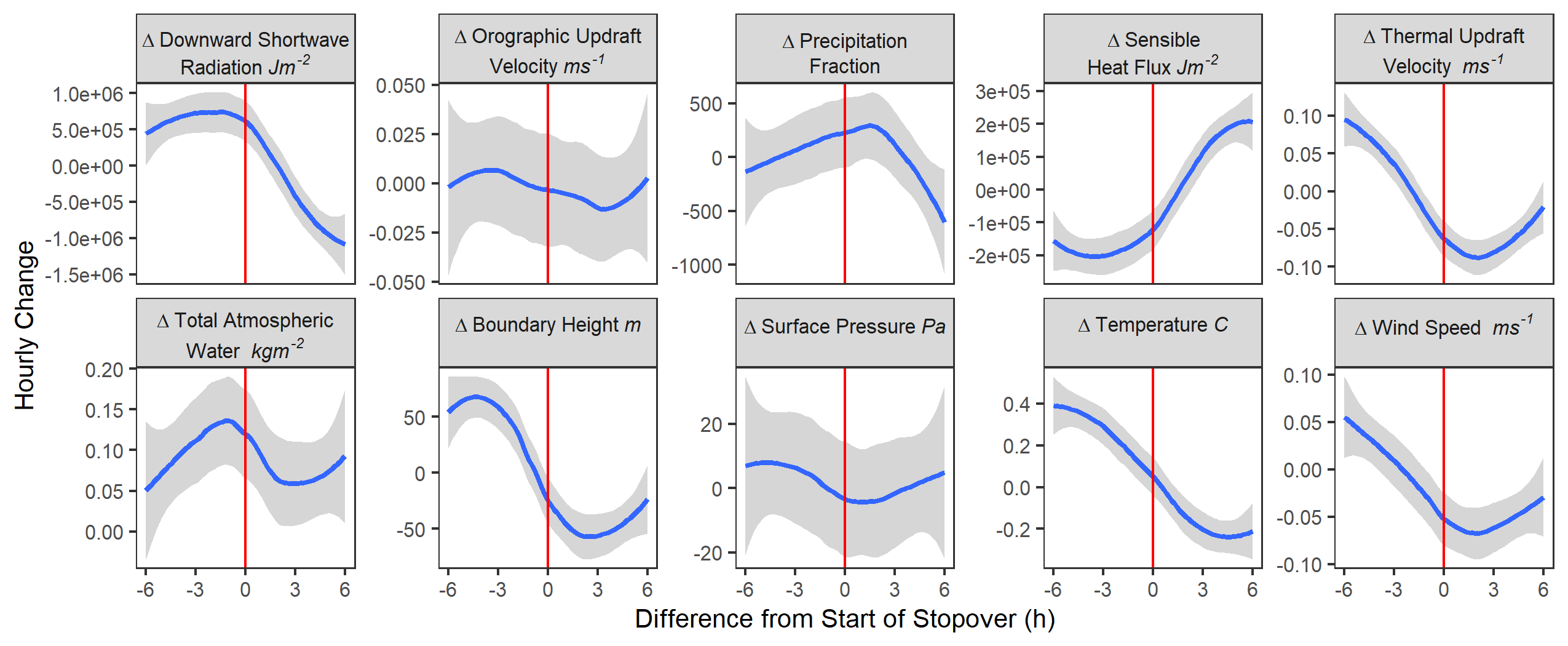


(c) Western Canada


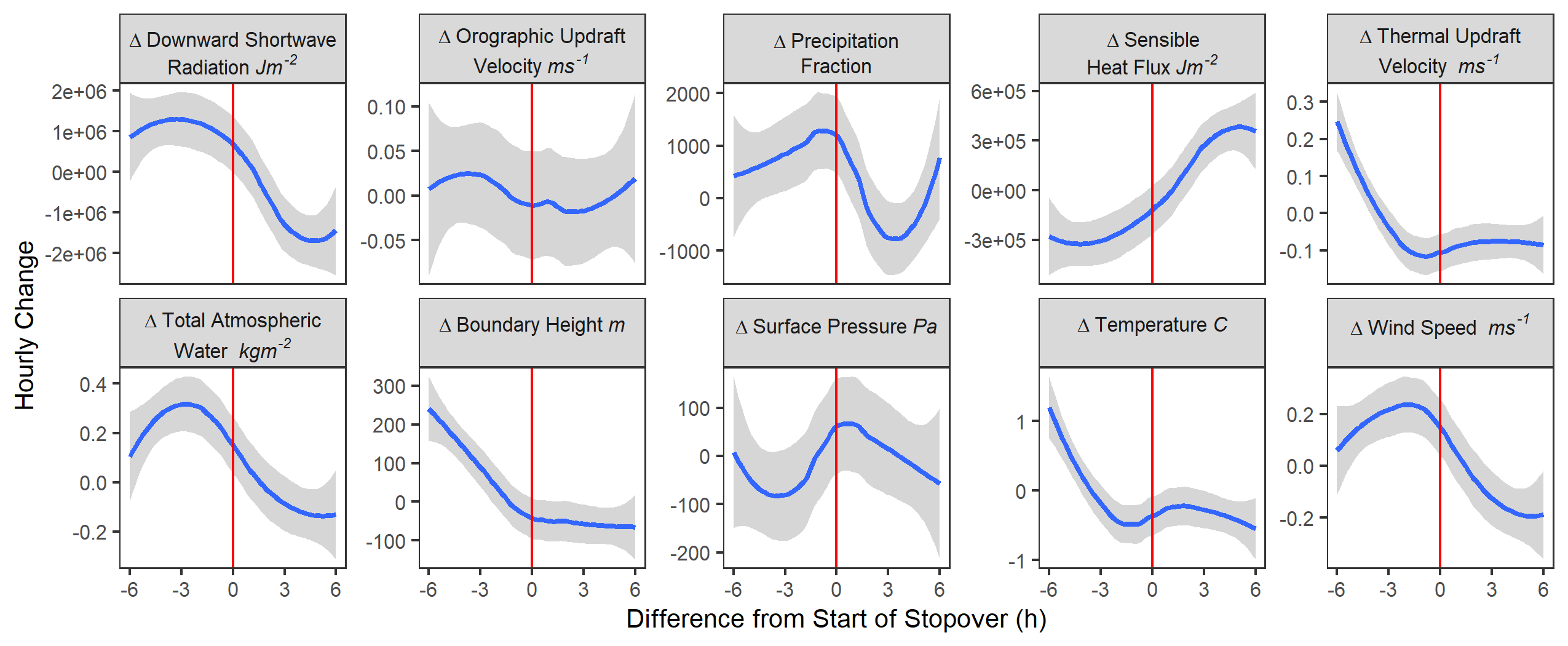


(d) Southern South America


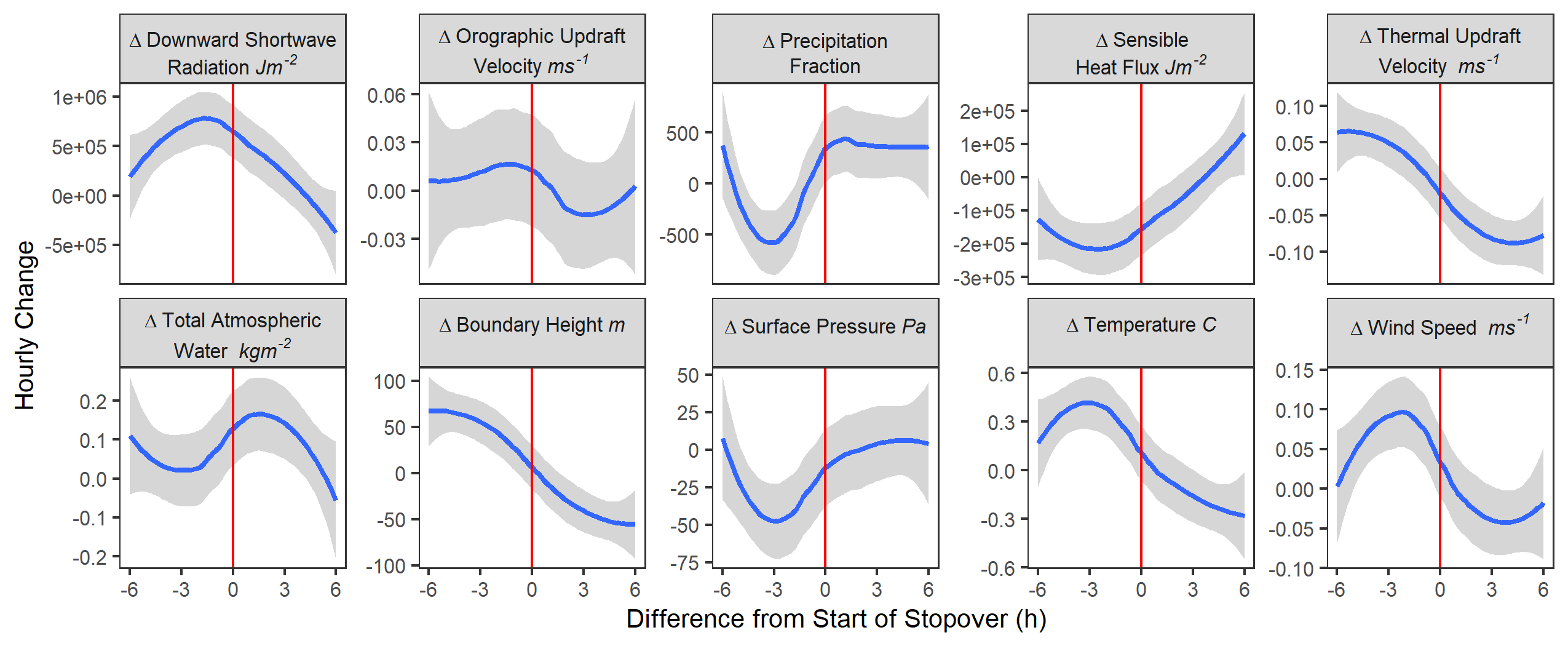


**Supplemental Figure 3.** Average weather conditions for each individual, relative to the start of identified stopovers (red line), by population. The y-axis represents the hourly change of the variable indicated in each plot’s title. Individuals in each population: (a) Southwest USA n = 14, (b) Central Canada n = 9, (c) Western Canada n = 6, and (d) Southern South America n = 5. Differences in responses across populations may be explained by unequal sample sizes, differing weather variable interactions associated with local climates, and stopovers used for feeding.

(a) Southwest USA


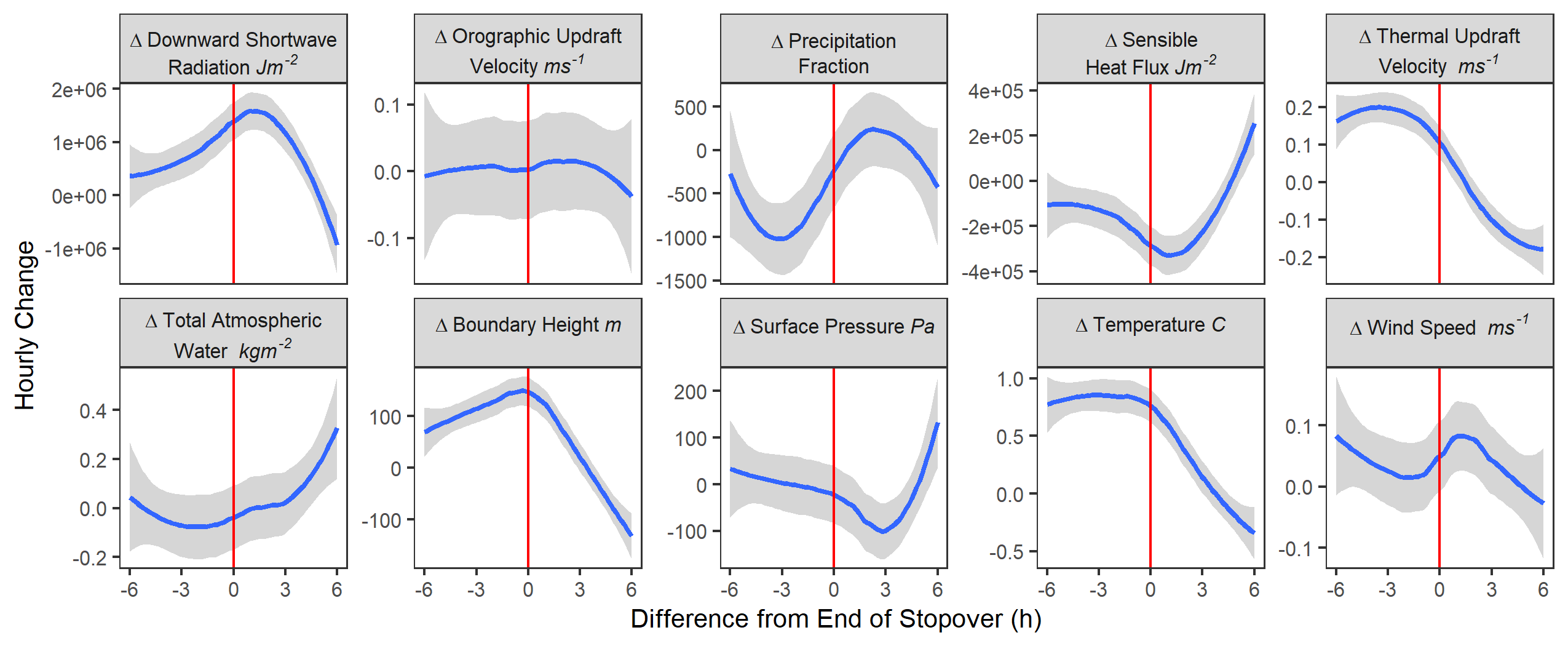


(b) Central Canada


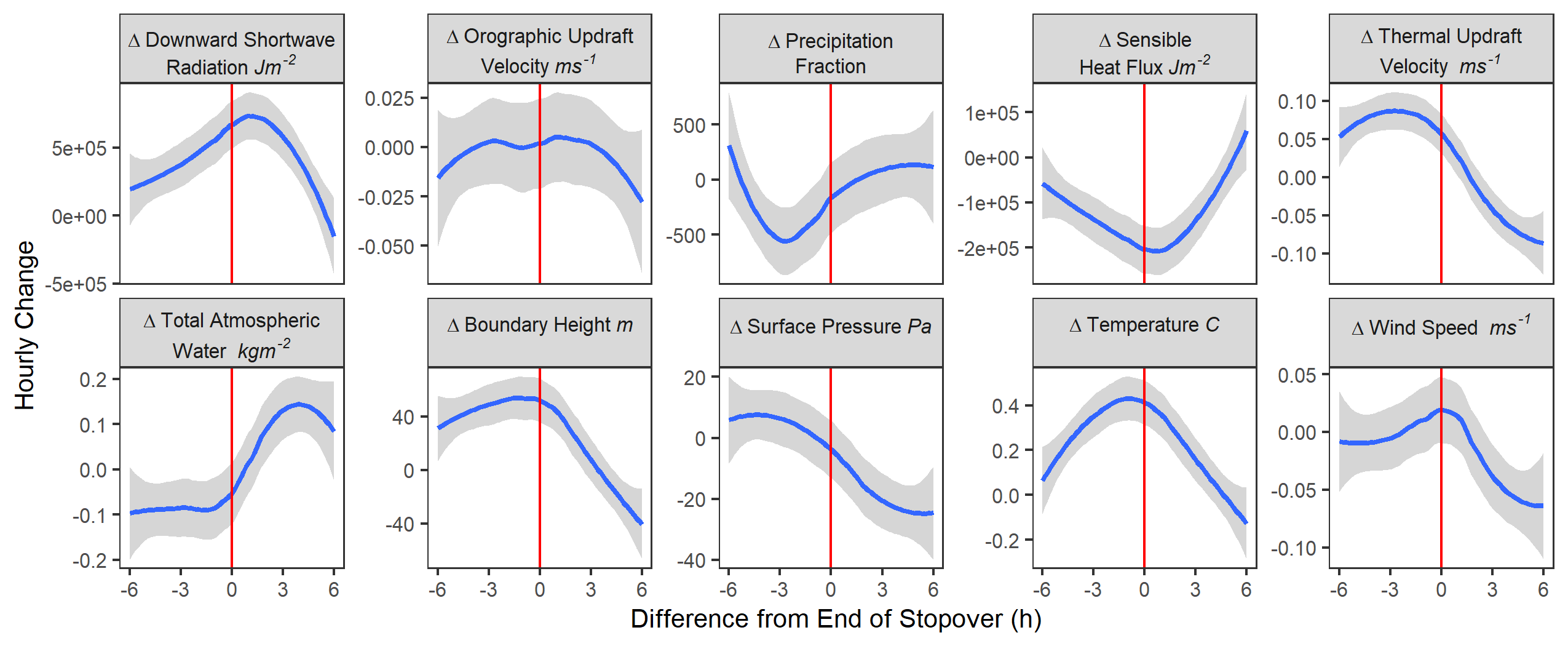


(c) Western Canada


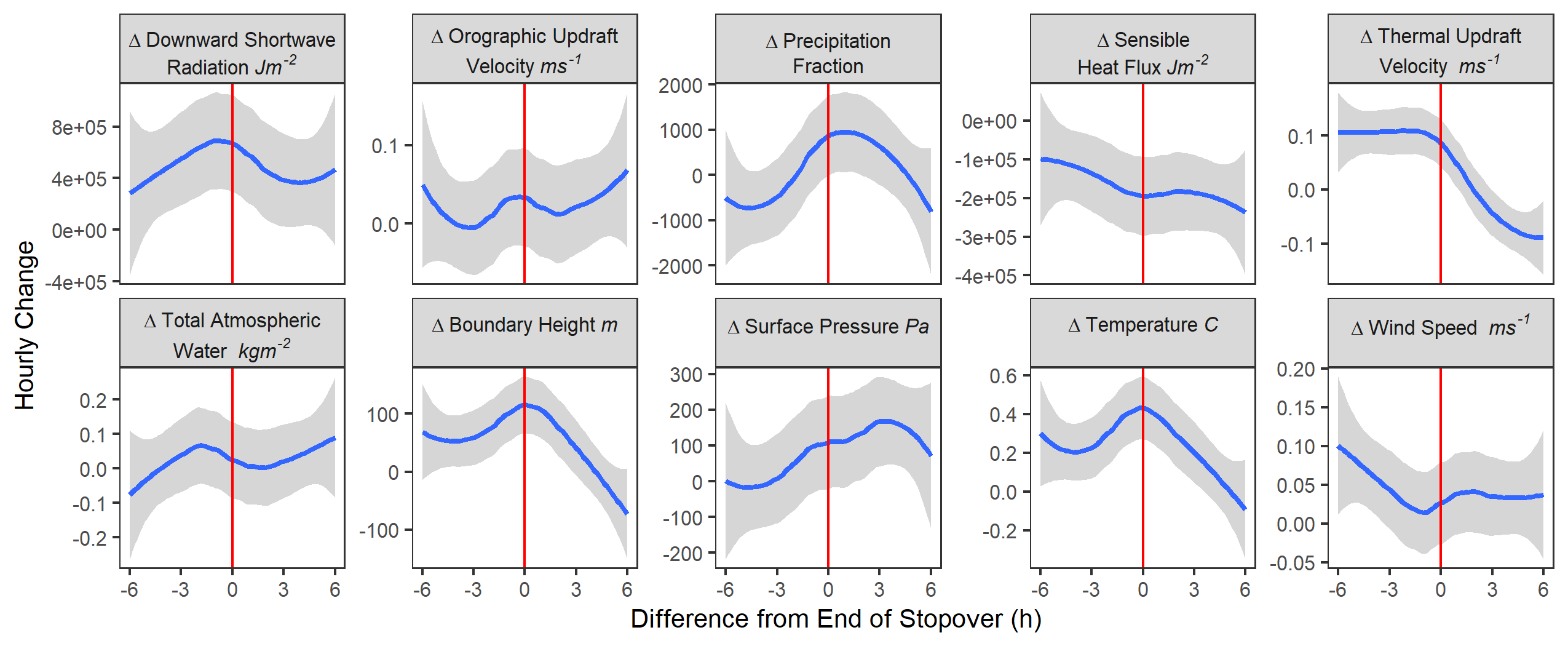


(d) Southern South America


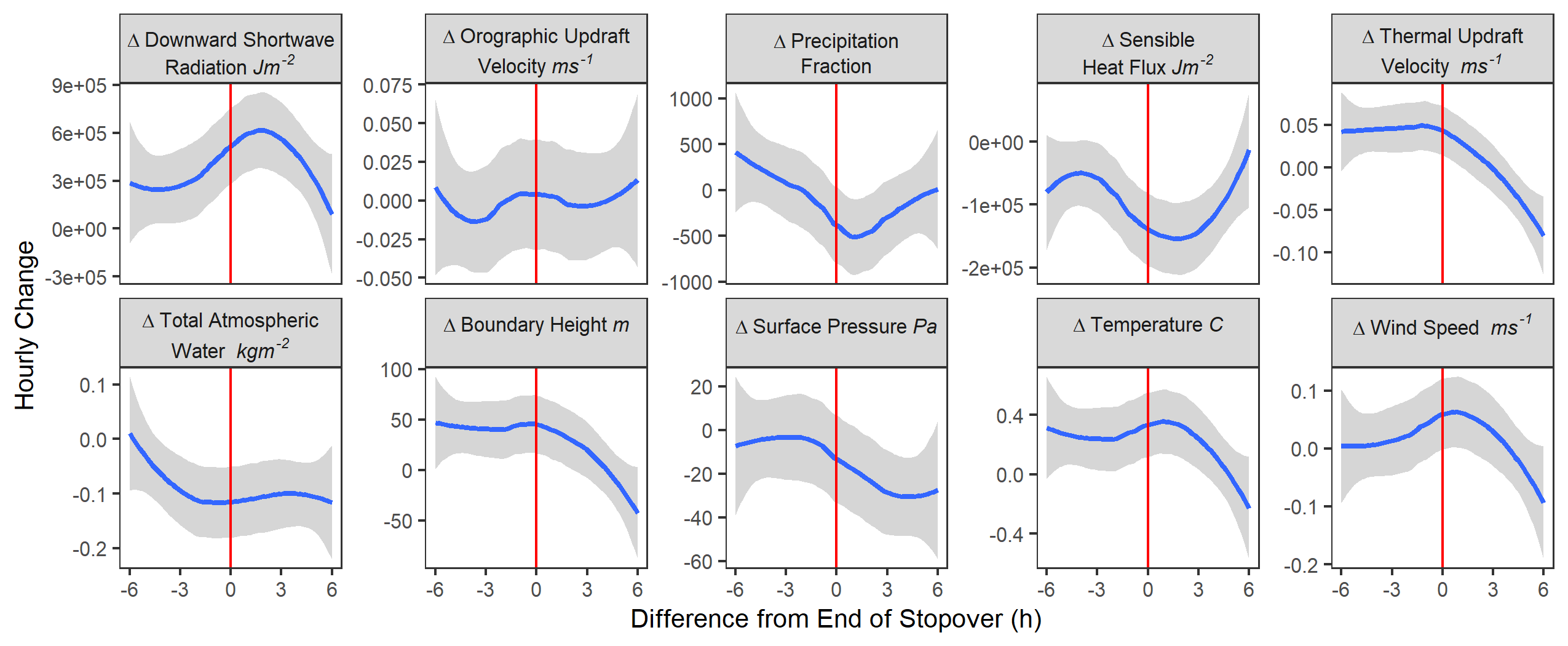


**Supplemental Figure 4.** Average weather conditions for each individual, relative to the end of identified stopovers (red line), by population. The y-axis represents the hourly change of the variable indicated in each plot’s title. Individuals in each population: (a) Southwest USA n = 14, (b) Central Canada n = 9, (c) Western Canada n = 6, (d) Southern South America n = 5. Differences in responses across populations may be explained by unequal sample sizes, differing weather variable interactions associated with local climates, and stopovers used for feeding.

**Supplemental Table 1.** Definitions of weather variables used and the rationale for including these variables. For variables that have units, units are provided in parentheses. Weather data were sourced from European Centre for Medium-Range Weather Forecasts (ECMWF) or Movebank.

| **Weather variable** | **Source** | **Definition** | **Rationale** |
| --- | --- | --- | --- |
| Air temperature (C) | ECMWF | Air temperature 2 m above the ground | Positively related to thermal soaring [1] |
| Boundary height (m) | ECMWF | The depth of air next to the earth’s surface which is most affected by the resistance to the transfer of momentum, heat, or moisture across the surface | Influence development of thermals [2,3,] |
| Downward shortwave thermal radiation (j m^-2^) | ECMWF | Amount of downward incident solar (shortwave) radiation at the earth’s surface | Influence development of thermals [2,3] |
| Precipitation fraction | ECMWF | The accumulated fraction of the model grid cell that was covered by large-scale precipitation | Negatively related to flight speed [4]  Increases likelihood of stopover [5] |
| Orographic updraft velocity (m s^-1^) | Movebank | The velocity of upward air movement caused when rising terrain forces air to higher elevations | Uplift source used during migration [6] |
| Sensible heat flux (j m^-2^) | ECMWF | Exchange of heat between the earth’s surface and the atmosphere through turbulent air motion, excluding any heat transfer resulting from condensation or evaporation (accumulated) | Used to calculate thermal updraft velocity [6]  Influence development of thermals [2,3] |
| Surface Air Pressure (Pa) | ECMWF | Atmospheric pressure at the earth’s surface | Passerines depart stopovers with decreasing surface pressure [7]  Positively related to migrant passage rate [8]  Detectable by avian species [9,10] |
| Thermal updraft velocity (m s^-1^) | Movebank | Velocity of thermal updrafts (air that rises as it is heated by the sun near the earth’s surface) | Uplift source used during migration [9] |
| Total atmospheric water (kg m m^-2^) | ECMWF | Total water in the entire atmospheric column (water vapor + cloud water + cloud ice) | Related to humidity and precipitable water, which are negatively related to thermal soaring [1, 56] |
| Windspeed (m s^-1^) | ECMWF | Calculated from wind u and v components | Positively related to thermal soaring [10] |

1. Mallon JM, Bildstein KL, Katzner TE. In-flight turbulence benefits soaring birds. Auk. 2015;133:79–85.

2. Stull RB. An introduction to boundary layer meteorology. Boston: Kluwer Academic Publishers. Springer 1988.

3. Duerr AE, Miller TA, Lanzone M, Brandes D, Cooper J, O’Malley K, Maisonneuve C, Tremblay JA, Katzner T. Flight response of slope-soaring birds to seasonal variation in thermal generation. Funct Ecol. 2015;29:779–90.

4. Vansteelant W, Bouten W, Klaassen R, Koks B, Schlaich A, Van Diermen J, Van Loon E, Shamoun-Baranes J. Regional and seasonal flight speeds of soaring migrants and the role of weather conditions at hourly and daily scales. J Avian Biol. 2015;46:25–39.

5. Goodrich LJ. Stopover ecology of autumn-migrating raptors in the central Appalachians. 2010.

6. Bohrer G, Brandes D, Mandel JT, Bildstein KL, Miller TA, Lanzone M, Katzner T, Maisonneuve C, Tremblay JA. Estimating updraft velocity components over large spatial scales: Contrasting migration strategies of golden eagles and turkey vultures. Ecol Lett. 2012;15:96–103.

7. Matthews SN, Rodewald PG. Urban forest patches and stopover duration of migratory Swainson’s thrushes. Condor. 2010;112:96–104.

8.﻿ Panuccio M, Dell’Omo G, Bogliani G, Catoni C, Sapir N. Migrating birds avoid flying through fog and low clouds. Int J Biometeorol. 2019;63:231–9.

9. Shamoun-Baranes J, Van Loon E, Alon D, Alpert P, Yom‐Tov Y, Leshem Y. Is there a connection between weather at departure sites, onset of migration and timing of soaring‐bird autumn migration in Israel? Glob Ecol Biogeogr. 2006;15:541–52.

10. Breuner CW, Sprague RS, Patterson SH, Woods HA. Environment, behavior and physiology: do birds use barometric pressure to predict storms? J Exp Bio. 2013;216:1982–90.

11. Laux CM, Nordell CJ, Fisher RJ, Ng JW, Wellicome TI, Bayne EM. Ferruginous Hawks Buteo regalis alter parental behaviours in response to approaching storms. J Ornithol. 2016;157:355–62.

**Supplemental Table 2.** Linear mixed model summary of the number of stops per migration by population and season. Model was fit using lme4 package version 1.1.23.

Linear mixed model fit by REML ['lmerMod']

Formula: stops ~ pop * Season + (1 | individual.local.identifier)

Random effects:

| Groups | Variance | Std. Dev. |
| --- | --- | --- |
| Individual.local.identifier | 2.913 | 1.707 |
| Residuals | 4.301 | 2.074 |

Number of obs: 174, groups: individual.local.identifier, 34

| Variable | B | SE | t |
| --- | --- | --- | --- |
| Intercept | 2.22289 | 0.56793 | 3.914 |
| Pop: Central Canada | 6.15947 | 0.96666 | 6.372 |
| Pop: Western Canada | -0.05406 | 1.11472 | -0.048 |
| Pop: South America | 1.95946 | 1.12601 | 1.740 |
| Season: Spring | -1.15619 | 0.45612 | -2.535 |
| Pop: Central Canada * Spring | 0.03522 | 0.80226 | 0.044 |
| Pop: Western Canada * Spring | 1.93769 | 0.99998 | 1.938 |
| Pop: South America * Spring | -0.19427 | 0.94655 | -0.205 |

**Supplemental Table 3.** Linear mixed model summary of the number of stops per migration by *total migration distance*. Model was fit using lme4 package version 1.1.23.

Linear mixed model fit by REML ['lmerMod']

Formula: stops ~ mig.dist + (1 | individual.local.identifier) + (1 | individual.local.identifier:population)

Random effects:

| Groups | Variance | Std. Dev. |
| --- | --- | --- |
| Individual.local.identifier | 0.9713 | 0.9855 |
| Individual.local.identifier:population | 1.6759 | 1.2946 |
| Residuals | 4.4190 | 2.1021 |

Number of obs: 174, groups: individual.local.identifier, 34; individual.local.identifier:pop, 34

Fixed effects:

| Variable | B | SE | t |
| --- | --- | --- | --- |
| Intercept | -2.8971172 | 0.9232498 | -3.138 |
| Mig.dist | 0.0016493 | 0.0002192 | 7.525 |
